# Sequential Application of Discrete Topographical Patterns Enhances Derivation of Functional Mesencephalic Dopaminergic Neurons from Human Induced Pluripotent Stem Cells

**DOI:** 10.1038/s41598-018-27653-1

**Published:** 2018-06-22

**Authors:** Kenneth K. B. Tan, Wallie Wee Meng Lim, Chou Chai, Marek Kukumberg, Kah Leong Lim, Eyleen L. K. Goh, Evelyn K. F. Yim

**Affiliations:** 1 Mechanobiology Institute Singapore, National University of Singapore, T-Lab, #05-01, 5A Engineering Drive 1, Singapore, 117411 Singapore; 2 Duke-NUS Medical School, 8 College Road, Singapore, 169857 Singapore; 3 Department of Research, National Neuroscience Institute, Singapore, 308433 Singapore; 4 Department of Physiology, National University of Singapore, Singapore, 117543 Singapore; 5 Department of Biomedical Engineering, National University of Singapore, EA-03–12, 9 Engineering Drive 1, Singapore, 117575 Singapore; 6 Department of Surgery, Yong Loo Lin School of Medicine, National University of Singapore, NUHS Tower Block, 1E Kent Ridge Road, Singapore, 119228 Singapore; 7 Department of Chemical Engineering, University of Waterloo, 200 University Avenue West, Waterloo, Ontario N2L 3G1 Canada; 8 Present Address: Institute of Medical Biology, 8A Biomedical Grove, #06–06 Immunos, Singapore, 138648 Singapore

## Abstract

Parkinson’s Disease is a progressive neurodegenerative disorder attributed to death of mesencephalic dopaminergic (DA) neurons. Pluripotent stem cells have great potential in the study for this late-onset disease, but acquirement of cells that are robust in quantity and quality is still technically demanding. Biophysical cues have been shown to direct stem cell fate, but the effect of different topographies in the lineage commitment and subsequent maturation stages of cells have been less examined. Using human induced pluripotent stem cells (iPSCs), we applied topographical patterns sequentially during differentiation stages and examined their ability to influence derivation yield and functionality of regionalized subtype-specific DA neurons. Gratings showed higher yield of DA neurons and may be beneficial for initial lineage commitment. Cells derived on pillars in the terminal differentiation stage have increased neuronal complexity, and were more capable of firing repetitive action potentials, showing that pillars yielded better network formation and functionality. Our topography platform can be applied to patient-derived iPSCs as well, and that cells harbouring LRRK2 mutation were more functionally mature when optimal topographies were applied sequentially. This will hopefully accelerate development of robust cell models that will provide novel insights into discovering new therapeutic approaches for Parkinson’s Disease.

## Introduction

Modelling human diseases using patient-specific stem cells can potentially impact the development of new therapeutic strategies for currently intractable neurodegenerative diseases such as Parkinson’s Disease (PD), but are limited by predictive and progressive cellular models that recapitulate late-onset disease phenotypes. PD is attributed to the selective death of ventral midbrain dopaminergic (DA) neurons in the substantia nigra, causing a reduced activity of dopamine in the nigrostriatal pathway^1^. With the advent of induced pluripotent stem cells (iPSC) technology, human pluripotent stem cells (PSCs) can be derived from patients and differentiated into disease-relevant cell types for cell modelling or therapy. Yet, cells derived from directed differentiation of human PSCs are mostly immature and often require long maturation process to establish functional properties that are robust^2,3^. The availability of physiological relevant *in vitro* models for PD is crucial to perform efficient screens, as well as for the discovery and development of therapeutics. Current efforts to accelerate drug screening protocols and streamline processing are dependent on the accessibility of fully functional human cell types. Thus, there is a critical need to enhance the differentiation as well as maturation of pluripotent stem-cell-derived cells *in vitro* which are robust in quantity and quality before their utility as disease models. It is thereby crucial to overcome this inadequacy that will hinder the ability to develop new, targeted interventions designed to treat PD.

Several studies have endeavoured toward enhancing the conversion efficiency of midbrain DA neurons, but these approaches have been constrained biochemically^4–7^. Biophysical signals can also affect stem cell proliferation, cell survival, as well as their propensity to differentiate into different cell types^8^. Indeed, several studies have demonstrated that the biophysical environment such as the topography that the cells adhere to, influence their response and can direct stem cell fate. It has been shown that micro- and nano-scale topographical surfaces induce changes in cell alignment, elongation, proliferation, polarization, migration, and gene expression^9,10^. For instance, cells cultured on gratings spontaneously elongate as well as align along the grating axis, leading to cells with a neuronal-like, highly polarized morphology^11–13^. Topographical cues may also be used to induce stem cell differentiation into different cell types. For example, gratings were shown to preferably direct mouse neural progenitor cells into dopaminergic neurons and reprogram mouse fibroblasts into DA neurons^13,14^. Meanwhile, pillars were also shown to accelarate neural differentiation^15^, affect polarization of neurons^16^, influence the morphology and growth directionality of dorsal root ganglion neurons^17^ and affect the branching and network formation^18^. Hence, as one of the effective approaches to utilize extracellular signals for cell fate decisions, substrate topography could provide an efficient strategy to enhance differentiation and improve cellular modelling of PD. To further contribute to research on cell attachment, proliferation, and differentiation, as well as developing next generation medical devices and implants, cell-substrate interactions at different stages of neuronal differentiation should be explored for applications towards the treatment of PD.

Here, we hypothesize that certain topographies when used in a temporal manner will enhance the derivation of mature and functional midbrain DA neurons from human pluripotent stem cells. We performed a 2-stage differentiation process and compared gratings and pillars in the maturation of midbrain DA neurons. We showed that the topographies enhanced the derivation and functionality of human midbrain DA neurons from healthy and patient-derived iPSCs. Our results will aid in the effort to produce robust quality DA neurons and provide novel insights into mechanisms underlying DA neuronal development, and ultimately discover new therapeutic approaches for this neurodegenerative disease.

## Results

### Differentiation of midbrain dopaminergic neurons on topographical cues

Induced pluripotent stem cells (iPSCs) derived from unaffected fibroblasts were differentiated into mesencephalic dopaminergic neurons on PDMS substrate chambers. The derived iPSCs were characterized to be pluripotent and karyotypically normal (Supplementary Figure 1). PDMS chambers were fabricated by soft lithography from PDMS molds (2 cm × 2 cm × 0.5 cm, L × W × H) in a 35 mm dish which can be fitted into the wells of a 6-well plate. The number of cells seeded on the patterns and the amount of differentiation media can thus be contained in the chamber and controlled with the minimal amount of growth factors. Cells can be fixed in the chambers and samples can be cut out for immunostaining.

**Figure 1:**
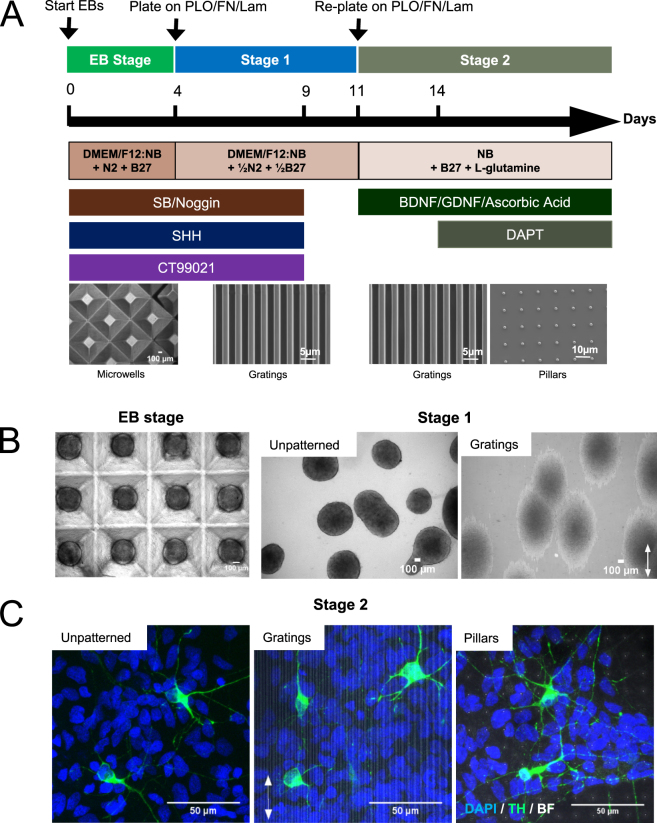
Controlled differentiation of human induced pluripotent stem cells (iPSCs) to midbrain dopaminergic neurons on patterned poly-dimethylsiloxane (PDMS). (**A**) Timeline of differentiation protocol of human iPSCs to dopaminergic neurons and the composition of the media at different stages. Modifications and optimization to the protocol based on dual SMAD inhibition method (Kirkeby *et al*. 2012) were made incorporating the use of topographical patterns for dopaminergic neuronal differentiation. The size of embryoid bodies was standardized on fabricated PDMS chambers consisting of microwells, whereupon they were harvested for a two-stage differentiation process on fabricated patterned chambers involving the expansion of neural progenitor cells (stage 1) and the terminal differentiation of neurons (stage 2). Concentrations of PLO/FN/Lam were optimized on the substrates for cell attachment. (**B**) Brightfield images of embryoid bodies in microwells and on patterned substrates at stage 1. Cells were observed to be aligned on the grating axis (arrow) at stage 1. Scale bar: 100 μm. (**C**) Immunofluorescence images of cells expressing tyrosine hydroxylase (TH), a dopaminergic neuron marker at stage 2 on patterned substrates. Scale bar: 50 μm. (EB: embryoid body; NB: neurobasal medium; PLO: poly-L-ornithine; FN: fibronectin; Lam: laminin; SHH: sonic hedgehog; BDNF: brain-derived neurotrohpic factor; GDNF: glial cell derived neurotrophic factor; TH: tyrosine hydroxylase; BF: brightfield).

To investigate whether cells cultured on different topographies would influence the derivation of midbrain dopaminergic neurons, iPSCs were differentiated into dopaminergic neurons with sequential application of topographical patterns in two stages. This involved the lineage commitment of neural progenitor cells on gratings (2 μm depth grating pattern with 2 μm ridge by 2 μm space) (stage 1) and the terminal differentiation of DA neurons on gratings or pillars (2 μm pillars with 12 μm pitch and 2 μm depth) (stage 2). The maturity and functionality of the derived neurons were compared at the end of stage 2. The patterns were replicated with good fidelity upon checking with scanning electron microscope (SEM) (Fig. 1A).

Human iPSCs were differentiated into dopaminergic neurons on PDMS replicas gratings or pillars for at least 21 days. Modifications and optimization to the protocol based on dual SMAD inhibition method^19,20^ were made incorporating the use of topographical patterns for dopaminergic neuronal differentiation. The size of embryoid bodies was standardized on fabricated PDMS chambers consisting of microwells 800 μm in size, whereupon they were harvested for a two-stage differentiation process on fabricated patterned chambers (Fig. 1A,B). Phase contrast images were taken at different stages of differentiation to keep track of cell interaction throughout the process (Supplementary Figure 2). Flow cytometry analysis of differentiated cells at the end of stage 1 (day 10) showed more cells expressing midbrain DA markers on the patterned substrates compared to unpatterned surface (Supplementary Figure 3). The dopaminergic neuron marker tyrosine hydroxylase (TH) was observed to be expressed on differentiated cells as early as 21 days of differentiation (stage 2), indicating successful derivation of dopaminergic neurons on the patterned substrates (Fig. 1C). Patient iPSCs harbouring LRRK2 mutation were also successfully differentiated on the patterned substrates with robust dopaminergic marker expression (Supplementary Figure 4).

**Figure 2:**
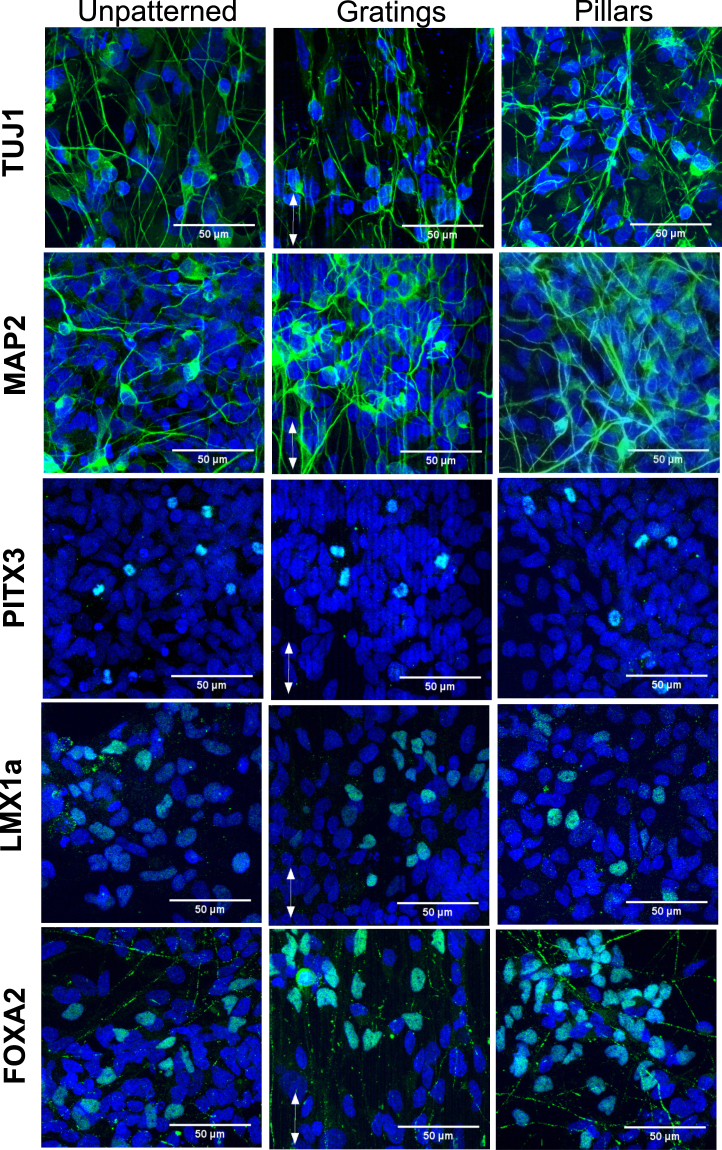
Immunostaining of dopaminergic neuronal markers on human induced pluripotent stem cell (iPSC)-derived cells on unpatterned, grating-, and pillar-patterned PDMS substrates. Beta-III tubulin (TUJ1), microtubule associated protein 2 (MAP2), pituitary homeobox 3 (PITX3), LIM homeobox transcription factor 1 alpha (LMX1a), forkhead box protein A2 (FOXA2).

Proteins that are involved in the maturation of midbrain dopaminergic neurons were further evaluated by immunofluorescence staining on patterned substrates (Fig. 2). Differentiated neurons were immunostained with antibodies for beta-III tubulin (TUJ1), microtubule-associated protein 2 (MAP2), pituitary homeobox 3 (PITX3), LIM homeobox transcription factor 1 alpha (LMX1a), and forkhead box protein A2 (FOXA2). These markers were expressed robustly in neurons grown on gratings and pillars. The presence of these markers indicated that the derived neurons were likely to be matured midbrain dopaminergic neurons (Fig. 2).

### Analysis of differentiation of midbrain dopaminergic neurons on topographical cues

**Figure 3:**
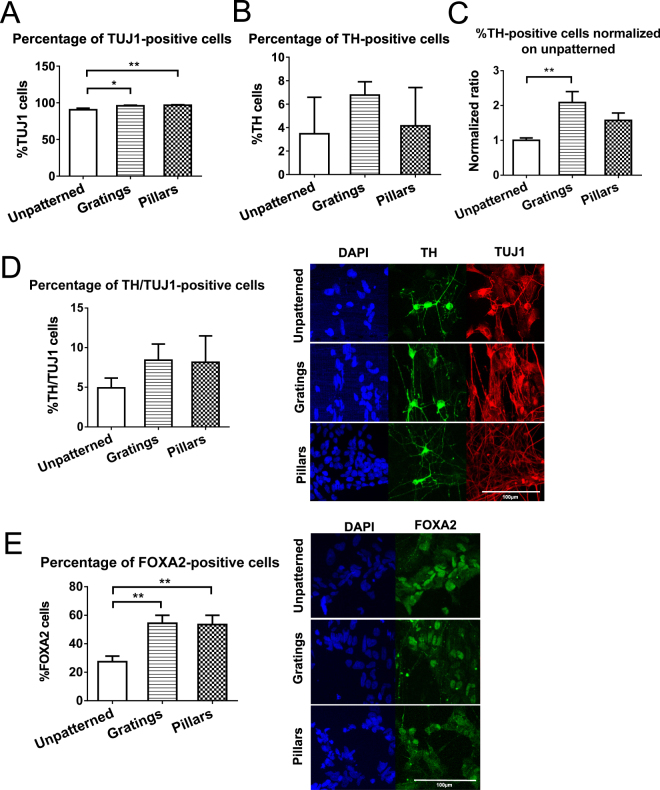
Quantification and data analysis of stained cells on patterned and unpatterned substrates after 21 day differentiation. (**A**) Quantification and data analysis of TUJ1-positive (neuronal) cells after 21 day differentiation on unpatterned, gratings and pillars PDMS substrates. Both gratings and pillars have higher percentages of TUJ1-positive cells than unpatterned (n = 5). (**B**) Quantification and data analysis of TH-positive (dopaminergic) cells after 21 day differentiation on unpatterned, gratings and pillars PDMS substrates (n = 5). (**C**) Percentage of TH-positive cells normalized on unpatterned substrates for each experiment. Gratings has about two-fold increase in TH-positive cells than unpatterned (n = 5). (**D**) Quantification and data analysis of TH/TUJ1 cells on unpatterned, gratings, and pillars PDMS substrates (n = 5). (**E**) Quantification and data analysis of FOXA2-positive cells on unpatterned, gratings, and pillars PDMS substrates. Gratings and pillars have more cells expressing FOXA2 than unpatterned (n = 3). All data are represented as data ± SEM of n independent experiments. At least five fields of view were taken for analysis and over 200–1000 cells were counted for each sample. *p < 0.05 **p < 0.01 compared to unpatterned control. Scale bar, 100 μm. (TUJ1: beta-III tubulin; TH: tyrosine hydroxylase; FOXA2: forkhead box A2).

To assess the effect of topographical cues on the derivation of midbrain dopaminergic neurons, quantification and image analyses were performed on cells stained with dopaminergic neuronal markers. All image analyses were performed at day 21 of differentiation (stage 2). Image analysis performed on the topographical substrates showed that the percentage of TUJ1+ neuron-specific cells were significantly higher on both the gratings (96.0 ± 0.9% of total cells; *p* < 0.05) and the pillars (96.8 ± 0.6%; *p* < 0.01), in comparison to the unpatterned control (90.6 ± 2.1%) (Fig. 3A). The average percentage of cells expressing the dopaminergic marker TH was highest on gratings (6.77 ± 1.1%) amongst the three substrates at day 21 (Fig. 3B), and had about a 2-fold increase of TH + cells when compared to unpatterned (*p* < 0.01) (Fig. 3C). Cells differentiated on pillars also contained more TH + cells than on the unpatterned control, with about 1.5 fold increase in TH+ cells (Fig. 3C).

The proportion of TH+ expressing cells among the TUJ1+ immature neuron sub-population (TH+/TUJ1+) was observed to be higher in gratings (8.42 ± 2.0%) and pillars (8.15 ± 3.3%) than the unpatterned controls (4.92 ± 1.2%), although there were no significant differences (Fig. 3D). Differentiation on gratings and pillars produced more cells that express FOXA2 (marker for midbrain dopaminergic) than the unpatterned control (27.5 ± 3.9%), with 54.4 ± 5.6% (*p* < 0.01) on gratings and 53.5 ± 6.5% (*p* < 0.01) on pillars (Fig. 3E). The percentage of TH+ cells that also express FOXA2 (FOXA2/TH) was also increased in gratings and pillars than the unpatterned controls, although there were no significant differences (Supplementary Figure 5).

### Topographical cues affect the length and neuronal complexity in iPSC-derived dopaminergic neurons

**Figure 4:**
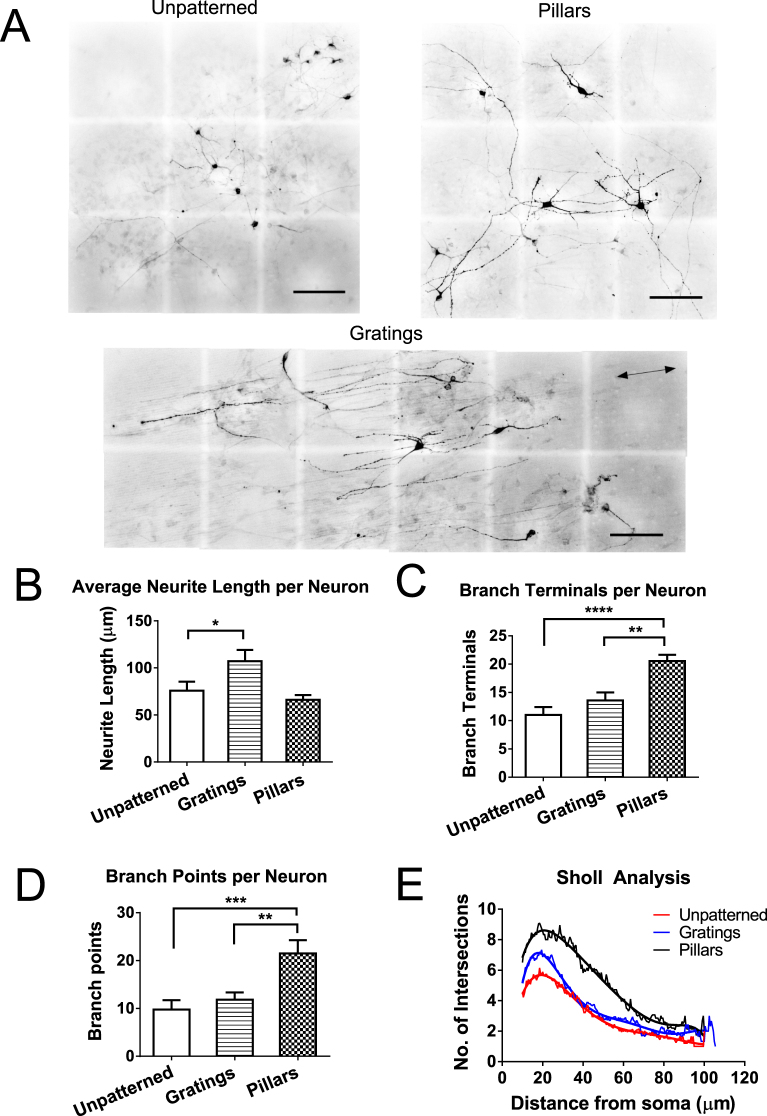
Morphology of TH neurons on patterned and unpatterned substrates after 21 day differentiation. (**A**) Representative images of human induced pluripotent stem cell (iPSC)-derived TH neurons on unpatterned, gratings and pillared PDMS substrates. Images were digitally stitched from overlapping fields of view to capture the entire length of neurites. Cells on gratings were aligned on the gratings axis (arrows). Scale bar: 100 μm. (**B**) Average neurite length per neuron. TH neurons were more elongated when differentiated on gratings than pillars and unpatterned control. (**C**) Number of terminals per neuron. (**D**) Number of branch points per neuron. (**E**) Sholl analysis. TH neurons have significantly more branching and increased neuronal complexity when differentiated on pillars than gratings and unpatterned control. All data are represented as data ± SEM of three independent experiments with over 30 TH-positive neurons analyzed on each pattern. *p < 0.05 **p < 0.01 ***p < 0.001 ****p < 0.0001.

To determine whether topographical cues affect the morphology and distribution of the differentiated dopaminergic neurons, TH+ neuronal cells were analysed on these patterns. As maturation of the differentiated cells are correlated with longer neurites, the average neurite length per neuron was quantified. The neurites were significantly more elongated when differentiated on gratings (average neurite length = 107.1 ± 11 μm; *p* < 0.05) as compared to the unpatterned control (average neurite length = 75.8 ± 9 μm) (Fig. 4A,B). As increased dendritic arborization would allow synaptogenesis to be formed between cells, the branching of the neurites was analysed as well. The number of branch terminals and branch points for each neuron were quantified to determine its branching morphology. TH+ neurons differentiated on pillars have more branch terminals and branch points compared to the unpatterned surface (86% and 170% more, respectively) and gratings (51% and 93% more, respectively) (*p* > 0.01). Neuronal complexity was also analysed by Sholl analysis on TH+ neurons (n = 30), which quantifies the number of neurite intersections for concentric circles of increasing radius spaced 10 μm apart centred on the soma (Fig. 4E). Neurons differentiated on pillars were more complex than those differentiated on the unpatterned surface and gratings, as indicated by the increased number of neurite intersections and the right-ward shift of the Sholl profile (Fig. 4E).

### Electrophysiology properties of iPSC-derived neurons on topographical cues

**Figure 5:**
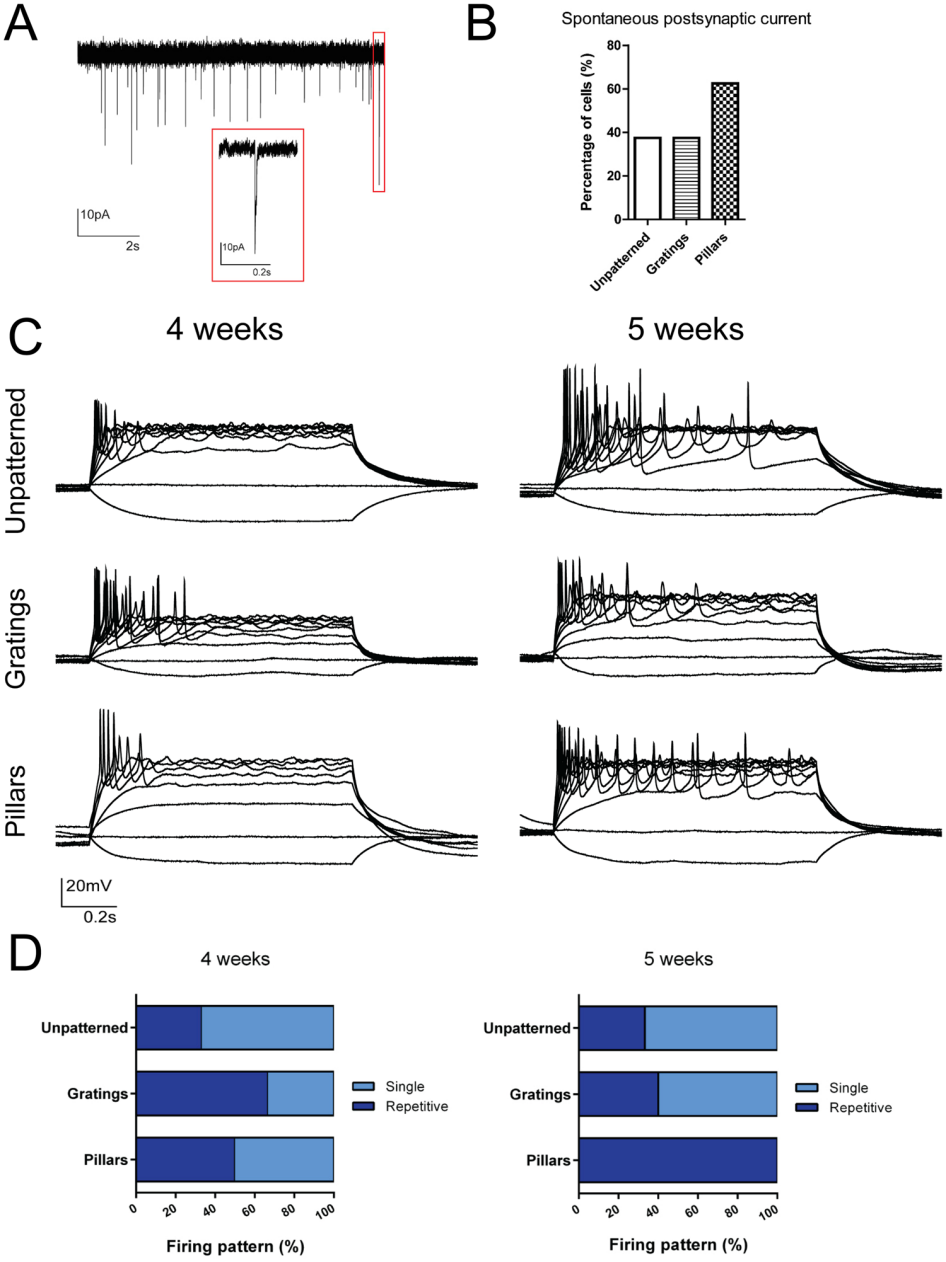
Electrophysiology properties of human induced pluripotent stem cell (iPSC)-derived neurons on unpatterned, grating-, and pillar-patterned PDMS substrates. (**A**) Representative trace shows spontaneous synaptic activity detected as early as 4 weeks post differentiation. Scale bar: 10 pA (vertical) and 2 s (horizontal). Inset (red box) scale bar: 10 pA (vertical) and 0.2 s (horizontal). (**B**) The percentage of cells having spontaneous postsynaptic current was higher in neurons differentiated on pillars. (**C**) Representative tracings of firing patterns on substrates at 4 and 5 weeks post-differentiation. Neurons were capable of firing repetitive action potential as a response to current injection as early as 4 weeks post-differentiation. (**D**) Percentage of differentiated neurons capable of repetitive firing at 4 and 5 weeks post-differentiation. More neurons on pillars were capable of repetitive firing than unpatterned and gratings at 5 weeks. 5 to 9 neurons were recorded from three independent experiments for each pattern.

To ascertain the differentiated neurons on the substrates were mature and functional, we performed patch clamping on the derived neurons to determine their electrophysiology properties. Cells were transduced with lentivirus expressing GFP driven by synapsin promoter to identify neurons in a heterogeneous cell population. Spontaneous synaptic activity was detected on neurons differentiated on patterned substrates, indicating the neurons were mature and functional as early as 4 weeks post-differentiation (Fig. 5A). Neurons differentiated on pillars showed 20% more cells with spontaneous postsynaptic current activity, in comparison to those on unpatterned and gratings (Fig. 5B). Action potentials in response to current injections could be detected as early as 4 weeks post-differentiation, with more frequent repetitive firing occurring at 5 weeks post-differentiation (Fig. 5C). Neurons that were differentiated on the patterned substrates were more capable of firing repetitive action potentials than those on unpatterned substrates, and almost all neurons that were recorded on pillars have repetitive action potentials at 5 weeks post-differentiation (Fig. 5D).

As a proof of concept study for using our topography platform on patient-derived iPSCs as well, we have also performed electrophysiology studies on iPSC-derived cells from PD patient harbouring LRRK2 mutation. When differentiated on gratings and pillars, the neurons were also shown to be more capable of firing repetitive action potentials at 5 weeks post differentiation than on unpatterned surface, suggesting increased maturity and functionality of these cells on patterns (Supplementary Figure 6).

## Discussion

In our study, we have developed novel methods to differentiate human pluripotent stem cells into ventral midbrain DA neurons in 2 stages via dual SMAD inhibition with sequential application of different topographical patterned substrates. This involved the lineage commitment of neural progenitor cells on gratings (stage 1), and the maturation of DA neurons on gratings or pillars (stage 2). We have chosen gratings and pillars as topographical patterns based on surmising of the existing literature from our group and other groups, as well as our unpublished observational studies^14,21^. We show that topographical cues are able to influence the differentiation of human induced pluripotent stem cells to midbrain dopaminergic neurons. Gratings were shown to increase the derivation of DA neurons from human induced pluripotent stem cells, suggesting that gratings are proficient for initial lineage commitment. In comparison, midbrain DA neurons that were derived on pillars in the maturation stage (stage 2) have increased branching and neuronal complexity. Electrophysiology studies also showed that cells differentiated on pillars in stage 2 were more capable of firing repetitive action potentials and have spontaneous postsynaptic activity, indicating that they were more mature and functional. Therefore, we suggest the use of gratings for initial lineage commitment of midbrain DA neurons, followed by re-plating the cells on pillars to promote neuronal function. We demonstrate that pillars can enhance DA neuronal maturation, and are desirable topographic patterns to improve neuronal functionality.

In the pursuit to determine how topographical cues can affect neuronal differentiation, most studies have been performed on gratings^10,11,21^. Indeed, gratings have been shown to enhance neuronal differentiation^11^ as well as DA neuronal differentiation from mouse neural progenitor cells^14^, but most of these studies have focussed on the yield of neurons. Additionally, the neural developmental pathways of rodents do not always recapitulate development in humans. Most studies also utilize general neuronal markers (MAP2) or early markers (TUJ1) to infer neuronal maturity at the lineage commitment stage, without terminal differentiating these cells and performing electrophysiology studies. The differentiation of human pluripotent stem cells into subtype-specific neurons on topography has not been well-explored as further specification and maturation are required for the derived neurons. The starting cell type used for DA neuronal differentiation is also an important factor. Although neural stem/progenitor cells derived from non-mesencephalic region can be differentiated towards DA-like neurons, this process is incomplete with much lower yield^22^, suggesting that regional specification in the brain occurs early in development. Therefore, early exposure of patterning factors during development is crucial for ventral midbrain DA neuronal differentiation. Thus, pluripotent stem cells are considered major cell source candidates for *in vitro* derivation of regional subtype specific neurons. We have utilized human pluripotent stem cells and differentiated them into subtype-specific neurons on topography in our studies, inferring neuronal maturation and functionality by electrophysiology, and hence providing a more relevant system for PD studies.

The mechanism of topography-induced differentiation of neurons remains not clearly understood. However, our earlier studies using human mesenchymal stem cells (hMSC) showed that gratings may change focal adhesion signalling as well as cytoskeletal contractility, which helped with cell lineage commitment^23^. As focal adhesion kinase (FAK) is essential in mechanosensing, it is possible that gratings and pillars provide better focal adhesion sites for cell attachment, altering actomyosin cytoskeleton contractility and transducing tensional force to nucleus to induce differential gene expression^23–27^. Actomyosin contractility may also play a role in the maturation of neurons, as inhibiting contractility led to a reduction of the maturation marker MAP2^26^. Enhanced growth and alignment of neurites in gratings attributed to the depth-sensing ability of the neurites^28^ have also been suggested to promote neuronal differentiation. Gratings have been shown to increase the rate of human embryonic stem cell (hESC) differentiation into neural lineage during lineage commitment^11^. As the application of grating topography have been shown in these studies to facilitate neuronal lineage commitment, this is consistent with our observation in stage 1.

One of the objectives of this study is to investigate the effect of different topographies at the stages of differentiation and maturation. While gratings have been shown to enhance neuronal lineage commitment, we introduced and re-plated the cells on pillars, gratings, and the unpatterned control to examine topographical influence on maturation. In one study, neural differentiation was accelerated with Tuj1+ and TAU+ cells appearing at earlier times when ESC-derived neural precursors were cultured on nanopillars^15^. Pillars were also shown to affect polarization of neurons^16^, as well as influencing morphology and growth directionality of dorsal root ganglion neurons^17^. Our observation of increased neurite branching on pillar topography may be due to the efficient anchoring of neuronal cell bodies by the pillars, which allowed the neurites to grow freely and branch into the surrounding area to develop a neuronal network^18^. We showed that when the pillar topography was applied at the maturation stage, it will not only result in increased branching as observed in a previous study^21^, but also enhances the maturation and changes electrophysiological properties of the neurons. In comparison, cortical neurons differentiated on gratings have been observed to display less branching^29^. Further evaluation on different feature sizes of the patterns may be performed to determine the optimal topographical dimensions for DA neuronal differentiation.

Modelling many late-onset diseases relies on efficient differentiation methods as mutant cell lines may inherently differentiate less efficiently. The complete specification and derivation of human midbrain DA neurons from human PSCs require at least 4 weeks to develop mature physiological behaviours that efficiently engraft in animal models of PD^30,31^. Here we show that repurposing topographical patterns at certain stages of differentiation enhances the yield and quality of midbrain DA neurons as compared to unpatterned surfaces. Although some mature neuronal cell types can be obtained using long and extensive protocols for PSC differentiation, neurons with fetal features can still persist^32–34^. These iPSC-derived DA neurons may still be too immature to capture the aging-related hallmarks of the disease, analogous to PD patients who do not display disease symptoms earlier on in life. Several studies have reported PD cell phenotypes in iPSC-based disease models of PD, but the reported cell types are of undefined relevance to PD such as neural stem cells^35^ or early biochemical phenotypes in DA neurons without modelling the late-onset degenerative nature of the disease^36–38^. To improve these model systems, the need for enhanced cell maturity is necessary to recapitulate the pathology of human disease, as well as for developing faithful models of late-onset neurodegenerative diseases such as PD.

Functional evaluation of these neurons is important to determine proper neuronal function and their physiological states. In the brains, action potentials are key characteristics of mature neurons as opposed to neural progenitor cells, as the latter express low levels of voltage gated ion channels necessary for the formation of action potential^39,40^. These ion channels, along with other genes, are upregulated during neural progenitor cells’ differentiation towards neurons^39^. We show that cells derived on the topographical patterns express markers for maturity with increased neurite branching and were functionally more robust as compared to cells on non-patterned surfaces. We observed spontaneous synaptic activity as early as 4 weeks (compared to 10 weeks^41^). Neurons generated with our protocol also exhibited repetitive firing behaviour earlier (4 weeks) than other studies (50–77 days)^41–43^. In addition, more repetitive firing neurons were found on patterned surfaces. This suggests patterned surface can yield more mature and functional cells. Repetitive firings in substantia nigra DA neurons are important functional indicators for their role in behaviours such as learned responses to stimuli and spatial learning^44–46^. The iPSC-derived neurons in our analysis have repetitive firing of >3 Hz, which is similar to that of DA neurons *in vivo*^47,48^, thus providing a cellular model that better recapitulates DA neurons *in vivo*. The enhancements in repetitive firing behaviour and spontaneous synaptic activity were observed probably because gratings were used to differentiate neurons in stage 1, increasing the overall enhancement of the yield and maturation of neurons. We have also optimized EB formation in microwells to ensure that the EBs were uniform in size. In addition, the soft PDMS substrate used for differentiation may also play a role in enhancing neural differentiation^49^.

Disease-specific phenotypes *in vitro* have been reported for certain mutations in familial PD which may reflect the pathophysiology and developmental implications of these mutations. For instance, neurons that were differentiated from patient iPSCs carrying LRRK2 and PARK2 mutations have reduced neuronal complexity and aberrant morphology^50,51^. As we have shown that neurons differentiated on pillars promote dendritic arborization with increased branching and neuronal complexity, it would be interesting to determine whether topographical cues would delay the onset of morphological defects that will lead to pathophysiological manifestation of disease. As proof of concept, we showed that cells harbouring LRRK2 mutation on topographical patterns were more capable of firing repetitive action potentials as demonstrated in electrophysiology studies (Supplementary Figure 6), suggesting that they can be driven to be more functionally robust. Application of biophysical cues that would alleviate or perhaps revert affected cells to a normal phenotype could uncover novel strategies in treating PD. Further studies could also be performed to determine whether aberrant changes in topographical substratum in the substantia nigra may play a role in the degeneration of DA neurons.

## Methods

### Fabrication and replication of polydimethylsiloxane (PDMS) patterned substrates

Sylgard 184 Silicone Elastomer kit (Dow Corning, Michigan, USA) was used to fabricate PDMS molds and substrate chambers via soft lithography. For the PDMS mold, the Sylgard 184 polymer and the accompanying curing agent were mixed with a ratio of 5:1 and desiccated under vacuum for 15 min. PDMS molds (2 cm × 2 cm × 0.5 cm, L × W × H) were cut into size and surface-treated with perfluorodecyltrichlorosilane (FTDS) and 0.01% TritonX 100 (Sigma Aldrich). To fabricate substrate chambers, the polymer mixture with polymer and curing agent at a ratio of 10:1 was poured into silanized patterned or unpatterned PDMS molds in a 35 mm dish, desiccated for another 1 hr and placed into a 70 °C oven to cure. The silanized PDMS molds were then removed to form a cuboidal chamber. The molds were replicated at least three times after silanization and stored in the oven to ensure complete crosslinking before using for experiments.

### Preparation of substrates for *in vitro* differentiation studies of pluripotent stem cells

The substrate chambers were washed in absolute ethanol and sterilized under ultraviolet light. They were fitted into the wells of a 6-well culture plate, and air-plasma treated for 120 seconds at 29.6 W (Harrick Plasma, Ithaca, NY, USA). For electrophysiology studies, a thin layer of patterned substrate less than 1 mm was cut and fitted on glass coverslips in a 24-well plate for culture. The substrates were then washed in absolute ethanol and placed under ultraviolet light for 20 min. After coating with poly-L-ornithine (0.01% in DI H_2_O) overnight (Sigma-Aldrich, Missouri, USA), the substrates were washed twice with sterile water and coated with fibronectin (50 µg/ml) (Biological Industries, Israel) and laminin (50 µg/ml) (Life Technologies, California, USA) overnight before cell seeding.

### Skin fibroblasts from PD patients and unaffected controls

Skin fibroblasts from a 57 year old male PD patient carrying a LRRK2 G2019S mutation (ND29542), and age- and sex-matched unaffected control (AG04148; 56 year old male) were purchased from the Coriell Institute for Biomedical Research (NJ, USA). Fibroblasts were maintained in T-25 flasks in MEMα supplemented with 15% FBS (both from Life Technologies). Subculturing of cells was performed using 1× trypsin/EDTA solution (Sigma T-4174) with standard techniques.

### Culture of human induced pluripotent stem cells (iPSCs)

Human iPSCs were derived from fibroblasts using the method previously described^52^, and subsequently adapted to feeder-free culture. The pluripotent stem cells were expanded on hESC-Qualified Matrigel (Corning, California, USA) coated polystyrene culture plates with mTeSR 1 medium (Stemcell Technologies, Vancouver, Canada).

### Controlled differentiation of pluripotent stem cells to dopaminergic neurons on substrate topography

Differentiation of human iPSCs to midbrain dopaminergic neurons was performed as previously described^53^ with modifications for use on patterned substrates. A graphical representation of the protocol is shown in Fig. 1. Embryoid bodies (EBs) were formed using microwell chambers (AggreWell 800, Stemcell Technologies). About 1.5 × 10^6^ cells were seeded in a single microwell chamber with EB medium (1:1 DMEM/F12: neurobasal media, 1 × N2, 1 × B27, L-glutamine) supplemented with SB431542 (10 µM) (Cellagen Technology, California, USA), Noggin (200 ng/ml) (R&D Systems, Minneapolis, USA), SHH-C24II (200 ng/ml) (R&D Systems), and CT99021 (0.8 µM) (Cellagen Technology). Y-27632 (10 µM) (Tocris Bioscience, Bristol, UK) were added for the first two days. The EBs were incubated at 37 °C with 5% CO_2_ and 95% humidity for 4 days with daily medium change. At day 4, the aggregates formed were dislodged by carefully drawing up the medium and firmly pipetting it back into the middle and around the wells. Dissociated EBs were seeded on grating pattern of 2 μm depth with 2 μm ridge by 2 μm space, or unpatterned controls in NPC media (1:1 DMEM/F12: neurobasal media, 0.5 × N2, 0.5 × B27, L-glutamine) supplemented with SB431542 (10 µM) (Cellagen Technology), Noggin (200 ng/ml) (R&D Systems), SHH-C24II (200 ng/ml) (R&D Systems), and CT99021 (0.8 µM) (Cellagen Technology). Supplemented media change was performed at day 7, followed by un-supplemented NPC media change on day 9 and day 10.

### Terminal differentiation to dopaminergic neurons on substrate topography

At day 11, the cells were detached using Accutase (Stem Cell Technologies) and replated on either gratings (2 μm depth grating pattern with 2 μm ridge by 2 μm space), pillars (2 μm pillars with 12 μm pitch and 2 μm depth), or unpatterned controls coated with poly-L-Ornithine (0.01%) (Sigma-Aldrich), fibronectin (50 µg/ml) (Biological Industries) and laminin (50 µg/ml) (Life Technologies). Cells were plated in 50 μl droplets with 0.5 × 10^6^ cells in each PDMS chamber. Once cells have been attached to the bottom, medium is then added. DA media (neurobasal media with 1 × B27 and L-glutamine) was changed every other day supplemented with BDNF (20 ng/ml) (Life Technologies), GDNF (10 ng/ml) (Life Technologies), and ascorbic acid (200 µM) (Sigma-Aldrich). DAPT (2.5 µM) (Tocris Bioscience) was supplemented to the media beginning day 14.

### Immunofluorescence staining

Cells were fixed in the PDMS chambers for 20 min with 4% paraformaldehyde, blocked with 10% donkey serum (Merck Millipore, Germany) or 10% goat serum (Life Technologies), and permeabilized with 0.1% Triton-X. The PDMS substrate was cut into smaller pieces to be incubated overnight at 4 °C with primary antibodies diluted in 1% donkey serum or 1% goat serum (Supplementary Table 1). This was followed by incubation with appropriate secondary antibodies for 1 hr. The secondary antibodies that were used included goat anti-rabbit Alexa Fluor 488, goat anti-mouse Alexa Fluor 546 (Life Technologies), or donkey anti-goat 488 (Abcam, England, UK) at the concentration of 1:500. Cells were counterstained with DAPI (Sigma-Aldrich) for 20 min and mounted with Prolong Gold antifade mounting media (Life Technologies). Cell images were taken with Leica DM IRM inverted microscope (Leica, Germany) or confocal microscope (Zeiss LSM710, Germany).

### Quantitative Image Analysis

Elongation of the neurons was calculated from the TH-positive population. At least five random, non-overlapping fields were taken at 40× objective for each sample from different independent experiments. Total cell counts in each field of the same sample were pooled to give a larger sample size for percentage counts and reduce bias from non-uniform distribution of cells. Total neurite length was analyzed by tracing each neurites emanating from the cell body using NeuronJ plugin in ImageJ^54^. The number of branch points and branch terminals were also analyzed after tracing. Sholl analysis was performed using the Sholl analysis plugin in ImageJ^55^.

### Scanning electron microscopy of replicated substrates

Samples were sputter-coated with platinum for 30 sec at 30 mA before scanning electron microscopy (SEM) was carried out in high vacuum at various magnifications.

### Virus transduction and whole-cell patch clamp electrophysiology

Electrophysiological recordings were performed on 4–5 week differentiated neurons that were transduced with a lentiviral vector carrying synapsin I promoter-driven green fluorescent protein (GFP) to identify neurons 4–5 days prior to recording. Cells were visualized using an inverted microscope (Carl Zeiss) and patched with 4–8 MΩ borosilicate glass pipettes (G150F-3, Warner Instruments) fashioned from a vertical puller (PC-10, Narishige). Pipettes were filled with K-gluconate internal solution containing (in mM): 120 K-gluconate, 9 KCl, 10 KOH, 3.5 MgCl_2_, 4 NaCl, 10 HEPES, 4 Na_2_ATP, 0.4 Na_3_GTP, 17.5 Sucrose, and 0.5 EGTA. Cells were bathed in an external solution containing (in mM): 125 NaCl, 23 NaHCO_3_, 10 glucose, 2.5 KCl, 0.8 NaH_2_PO_4_, 2.5 CaCl_2_, and 1 MgCl_2_. Osmolarity of the internal and external solutions was measured by an osmometer (VAPRO 5600, ELITechGroup), ranged around 290 and 310 mOsm/kg, respectively. After break-in, cells were clamped in current-clamp mode for recording. Action potentials (APs) were elicited by step-wise increment of 20 pA, starting from −20 pA to 160 pA for 1 second, and analyzed using the Clampfit software (Molecular Devices). Cells were categorized according to their AP firing pattern: single (a maximum of 1 AP per second) or repetitive (at least 3 AP per second). Spontaneous synaptic currents were recorded for two minutes at the resting membrane potential of the neurons measured right after break-in.

### Statistical analysis

Data was presented as the mean and standard error of mean where appropriate with all the experiments carried out in duplicates or more. Data analysis was performed by one-way analysis of variance (ANOVA), followed by post-hoc Dunnett’s test for multiple comparisons of at least three groups with Prism 6.0 (Graphpad Software Inc, California). *p* values of < 0.05 were considered statistically significant.

## Conclusion

Topographical modulation of stem cell differentiation into subtype-specific neurons may have significant implications in cell-based therapy for the treatment of PD. By showing here how cell interaction with biophysical cues influences DA neuronal differentiation, this will potentially accelerate the development of robust cell models which will aid in dissecting the complex pathological process of Parkinson’s Disease and ultimately discover novel therapeutic interventions for this disease.

## Electronic supplementary material

Supplementary Information
