# Supplementary material for: Sequential Application of Discrete Topographical Patterns Enhances Derivation of Functional Mesencephalic Dopaminergic Neurons from Human Induced Pluripotent Stem Cells
Source: Sci Rep. 2018 Jun 22;8:9567. doi: 10.1038/s41598-018-27653-1 (PMC6014983; doi:10.1038/s41598-018-27653-1)
Supplement: Supplementary file 1 — Supplementary Information [file 41598_2018_27653_MOESM1_ESM.pdf]

Sequential Application of Discrete Topographical Patterns Enhances Derivation of Functional Mesencephalic Dopaminergic Neurons from Human Induced Pluripotent Stem Cells

Kenneth K.B. Tan, Wallie Wee Meng Lim, Chou Chai, Marek Kukumberg, Kah Leong Lim, Eyleen L.K. Goh, Evelyn K.F. Yim

SUPPLEMENTARY MATERIALS AND METHODS

Skin fibroblasts from PD patients and unaffected controls

Skin fibroblasts from a 57 year old male PD patient carrying a LRRK2 G2019S mutation (ND29542) and age- and sex-matched unaffected control (AG04148; 56 year old male) were purchased from the Coriell Institute for Biomedical Research (NJ, USA). Fibroblasts were maintained in T-25 flasks in MEMα supplemented with 15% FBS (both from Life Technologies). Subculturing of cells was performed using 1× trypsin/EDTA solution (Sigma T-4174) with standard techniques.

Derivation of induced pluripotent stem cells (iPSCs)

Somatic reprogramming of fibroblasts was performed using a modification of the EBNA-1 based episomal reprogramming method described by Okita et al. [1]. The episomal vectors pCXLE-hOCT3/4-shp53-F, pCXLE-hSK, and pCXLE-hUL were purchased from Addgene (#27077, 27078, and 27080 respectively). Actively growing fibroblasts was dissociated using 1× trypsin/EDTA solution. Cells were counted and 6×10^5 cells were pelleted and resuspended in 100 µl Buffer R from the Neon™ Transfection System 100 µl Kit (Life Technologies, MPK10096). The cells suspension was mixed with 1 µg each of the 3 reprogramming vectors and loaded into a 100 µl Neon® Tip. Transfection was performed on the Neon electroporation device (Life Technologies MPK5000) using the following parameters: 1650V, 10 ms, 2 pulses. Transfected cells were distributed evenly into 3 wells of a 6-well plate (BD Falcon) and maintained in complete DMEM medium (DMEM high glucose #11960-044, 10% FBS, 1× GlutaMAX, 100 µM non-essential amino acid, 1 mM sodium pyruvate, 100 µM β-mercaptoethanol, all from Life Technologies) without antibiotics for 7 days with medium change every other day. On Day 7,

cells were dissociated using 1× trypsin/EDTA solution and counted. The subsequent steps differ from those described in the original Okita et al. protocol and represent our adaptation of the protocol for feeder-free iPSC derivation. Instead of seeding the dissociated cells on inactivated mouse feeder layer, 1×10^5 cells were plated per well of Matrigel (Becton-Dickinson #354277) coated 6-well plate in complete DMEM medium. The following day, the medium was replaced with a 1:1 mixture of complete DMEM and defined pluripotent stem cell medium mTeSR1 (StemCell Technologies #05850). Starting from the next day, medium was switched to mTeSR1. Medium change was performed every other day. When iPSC colonies reach about ~0.5 -1 mM in diameter (around 3 weeks post-transfection onward), they were manually picked under bright field microscopy and expanded. Passaging of iPSCs was performed by complete dissociation into single cells.

Immunofluorescence staining of iPSCs

Cells were cultured on glass coverslips, fixed in 4% paraformaldehye and incubated with the following primary antibodies: Klf4 (Stemgent 09-0021), Oct4 (Santa Cruz sc-5279, 1:200), Nanog (Abcam 80892, 1:200), Sox2 (Abcam ab59776, 1:200), Tra-1-81 (Millipore MAB4381, 1:100) and SSEA4 (Millipore MAB4304, 1:100). Alexa Flour 488 and Alexa Fluor 555 labelled donkey secondary antibodies (all from Life Technologies) against the appropriate host animal were used at 1:400 dilutions.

Karyotype analysis

Chromosomal G band analysis was performed by the Cytogenetics Laboratory, Department of Pathology and Laboratory Medicine, Kandang Kerbau Women's and Children's Hospital Pte Ltd, Singapore.

SUPPLEMENTARY REFERENCES

1. Okita K, Matsumura Y, Sato Y, Okada A, Morizane A, Okamoto S, Hong H, Nakagawa M, Tanabe K, Tezuka K, Shibata T, Kunisada T, Takahashi M, Takahashi J, Saji H, Yamanaka S. A more efficient method to generate integration-free human iPS cells. Nat Methods. 2011 May;8(5):409-12.

Supplementary Table 1. List of antibodies for staining

Antigen	Dilution	Source	Species
β-III-tubulin (TUJ1)	1:350	Covance	Mouse
LIM homeobox transcription factor 1, alpha (LMX1a)	1:500	Millipore	Rabbit
Microtubule associated protein 2 (MAP2)	1:500	Abcam	Mouse
Nuclear receptor related 1 protein (NURR1)	1:50	Abcam	Mouse
Pituitary homeobox 3 (PITX3)	1:50	R&D Systems	Rabbit
Tyrosine hydroxylase (TH)	1:500	Pel-Freez	Rabbit
FOXA2 (HNF-3β)	1:50	Santa Cruz	Goat

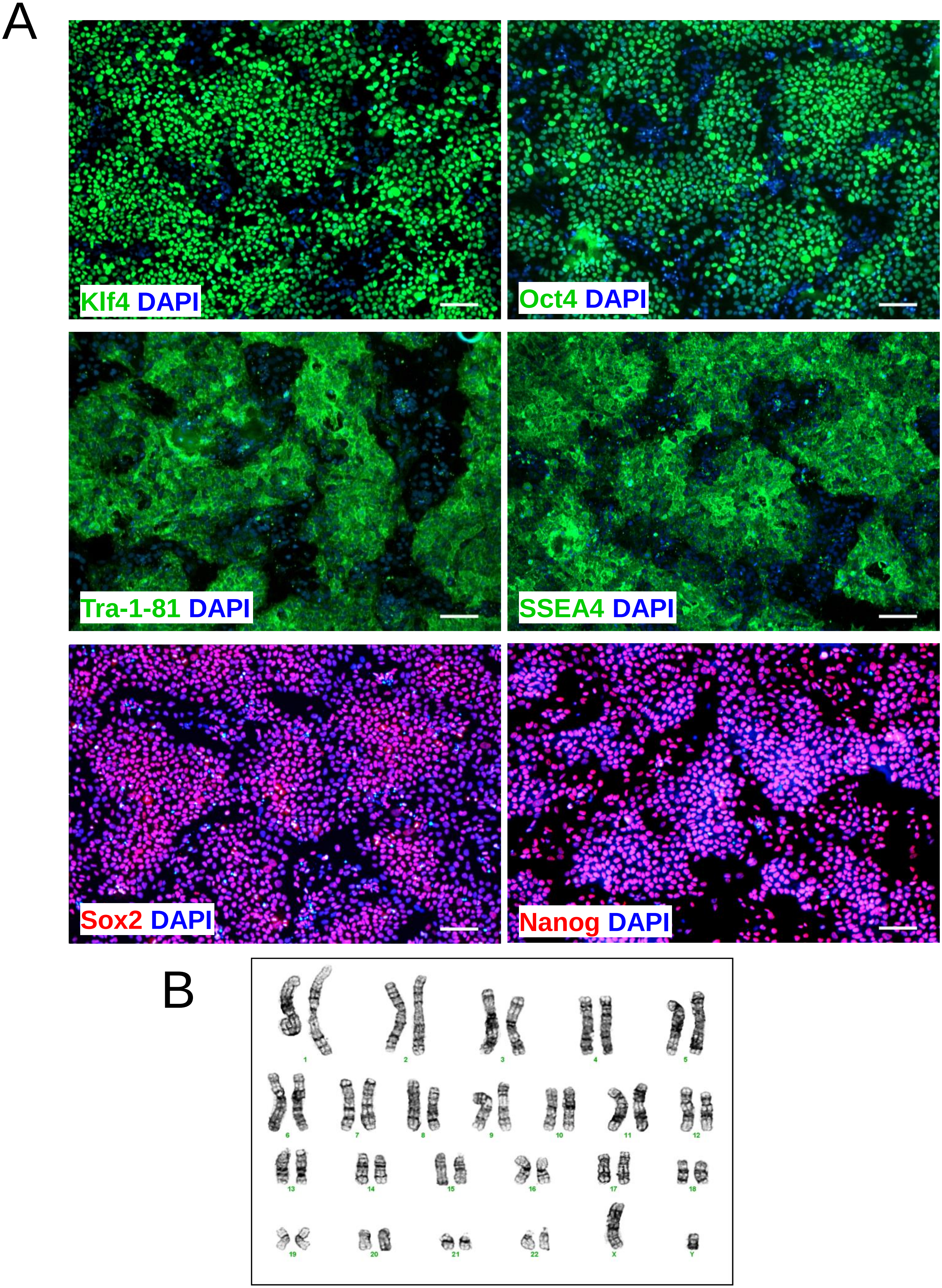
A
Klf4 DAPI
Oct4 DAPI
Tra-1-81 DAPI
SSEA4 DAPI
Sox2 DAPI
Nanog DAPI
B
1
2
3
4
5
6
7
8
9
10
11
12
13
14
15
16
17
18
19
20
21
22
X
Y

Supplementary Figure 1. Characterization of iPSCs derived from fibroblasts. (A) Immunostaining for pluripotency markers Klf4, Oct4, Tra-1-81, SSEA4, Sox2 and Nanog in iPSCs derived from AG04148 fibroblasts. Scale bar: 100 μm. (B) Chromosomal integrity of iPSCs was confirmed by karyotype analysis.

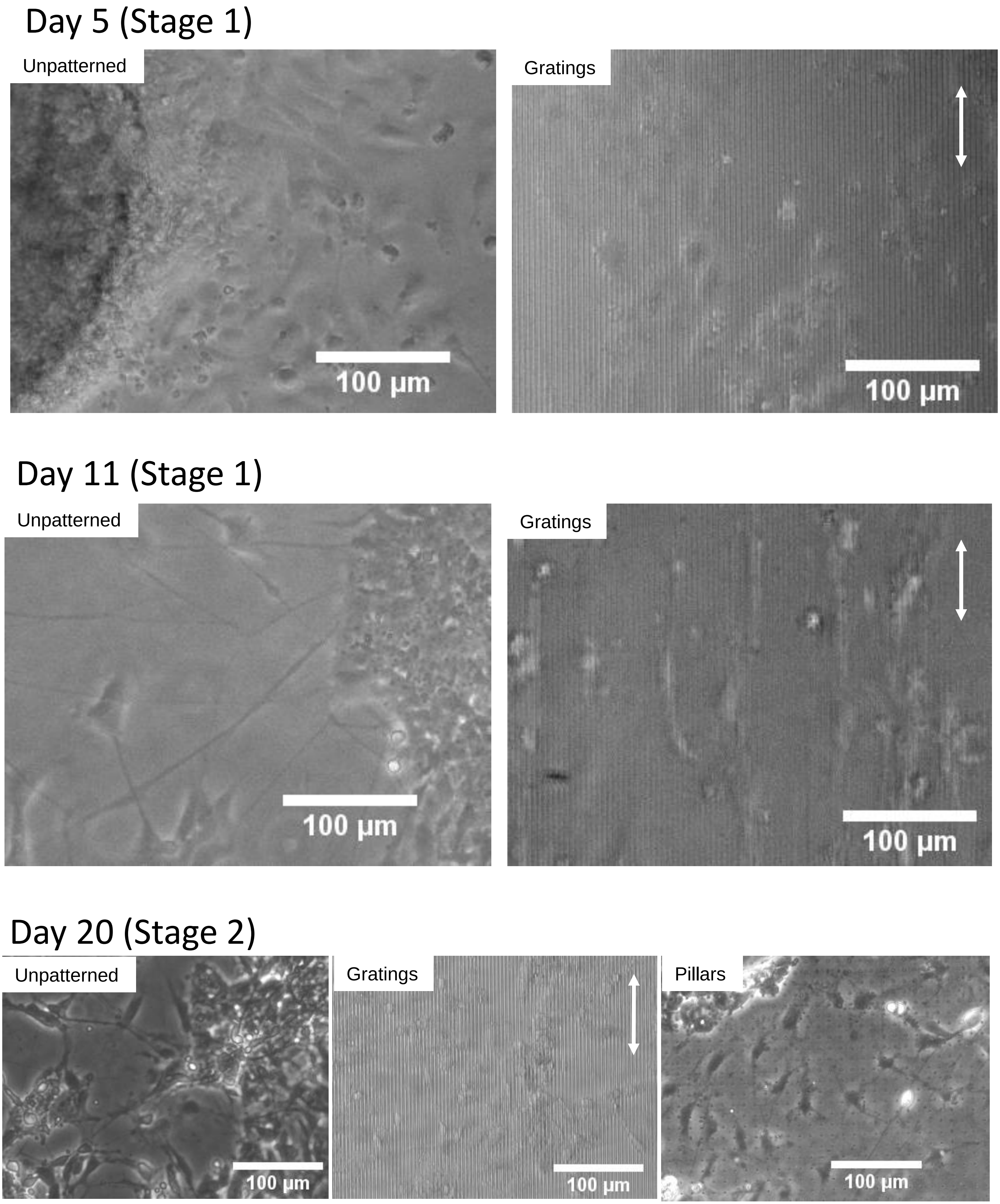

Supplementary Figure 2. Phase contrast images of cells on patterned substrates at stage 1 and stage 2. Scale bar: 100 µm

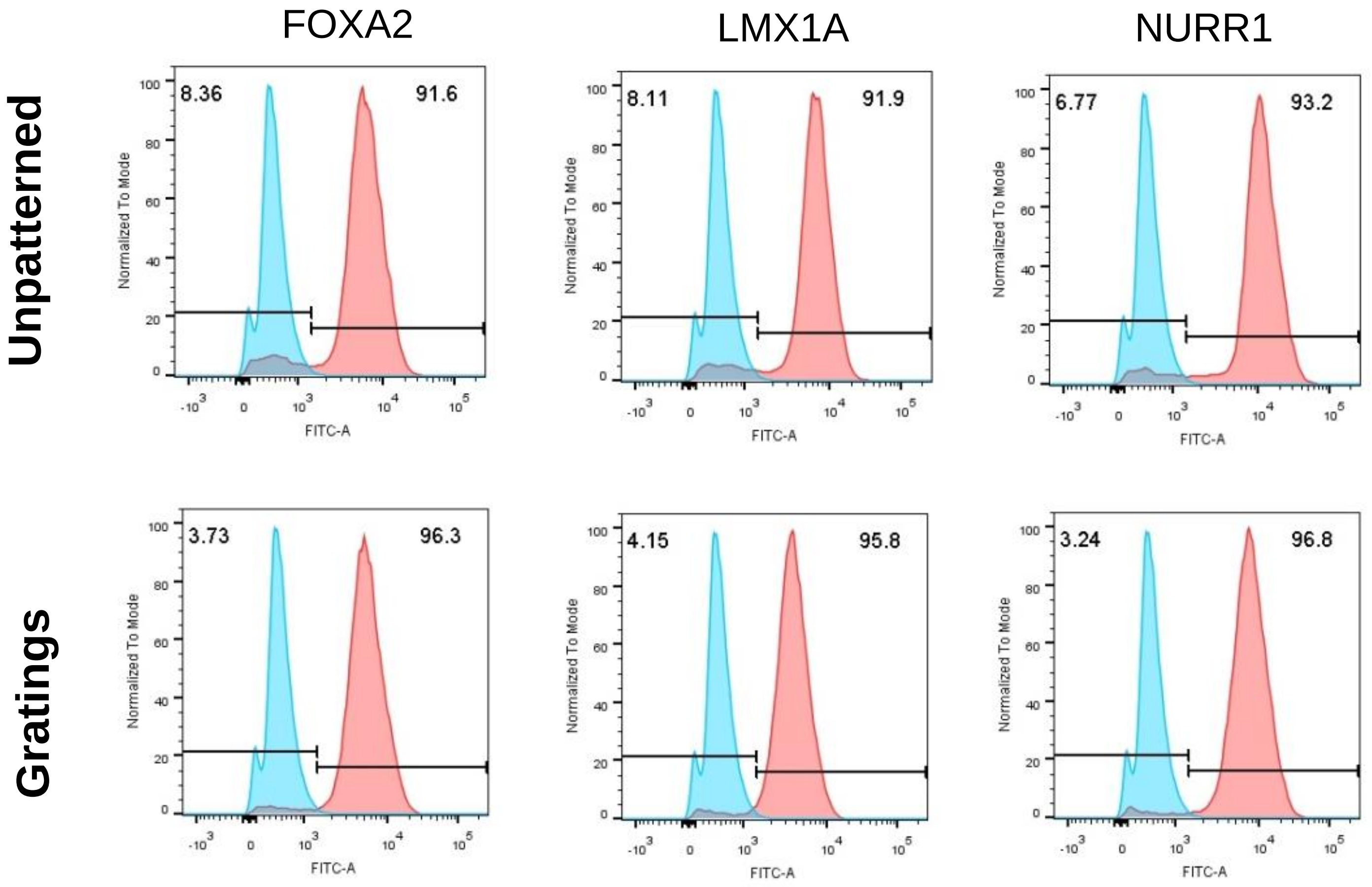

Supplementary Figure 3. Flow cytometry analysis of differentiated cells at the end of stage 1. Cells express more midbrain DA markers on patterned substrates. (FOXA2: Forkhead Box A2; LMX1a: LIM homeobox transcription factor 1, alpha; NURR1: nuclear receptor related 1 protein)

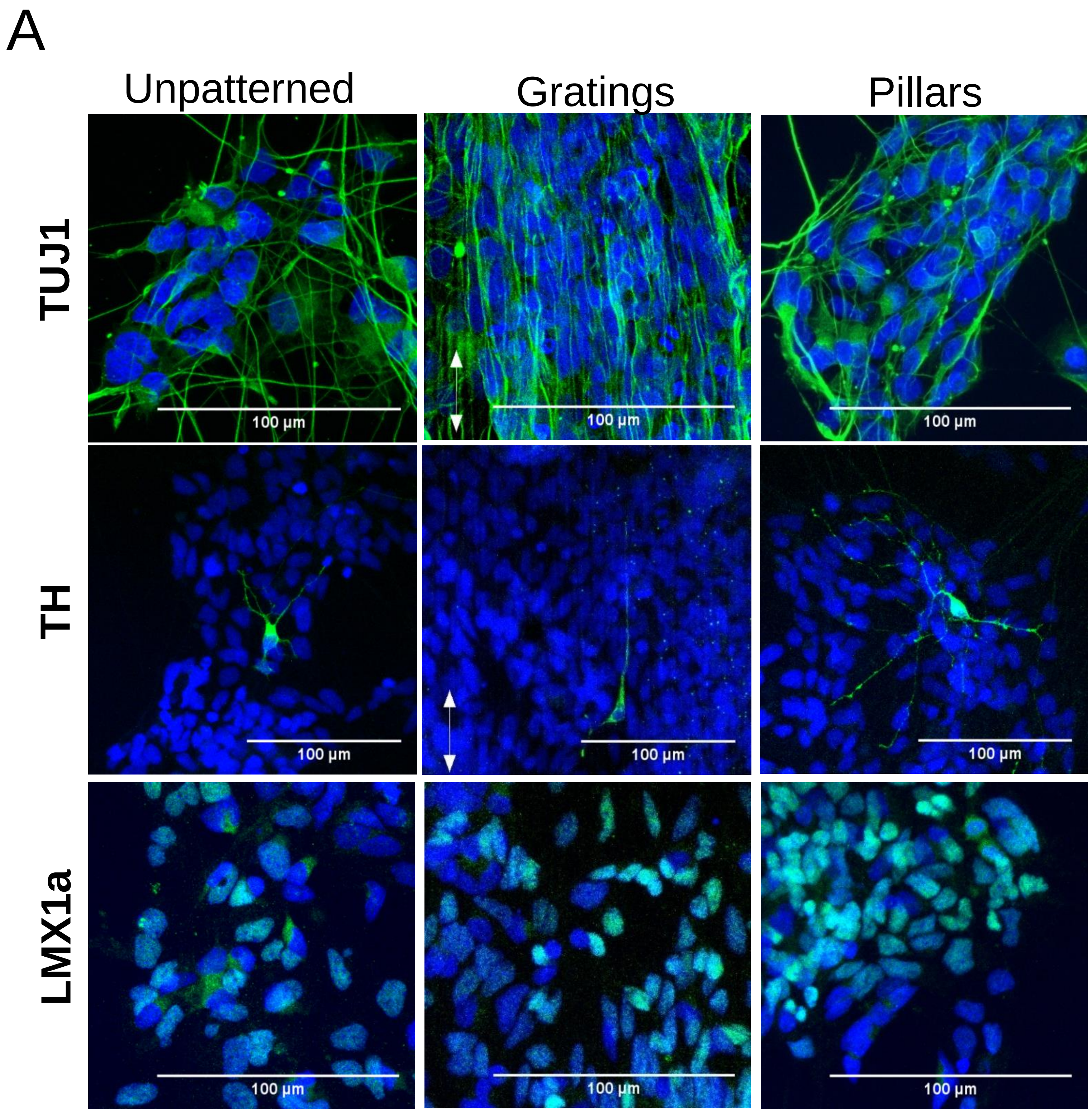

Supplementary Figure 4. Immunostaining of dopaminergic neuronal markers on human induced pluripotent stem cell (iPSC)-derived cells from PD patient with LRRK2 mutation on unpatterned, gratings, and pillars PDMS substrates. Beta-III tubulin (TUJ1), microtubule associated protein 2 (MAP2), pituitary homeobox 3 (PITX3), LIM homeobox transcription factor 1 alpha (LMX1a), forkhead box protein A2 (FOXA2). Scale bar: 100 μm

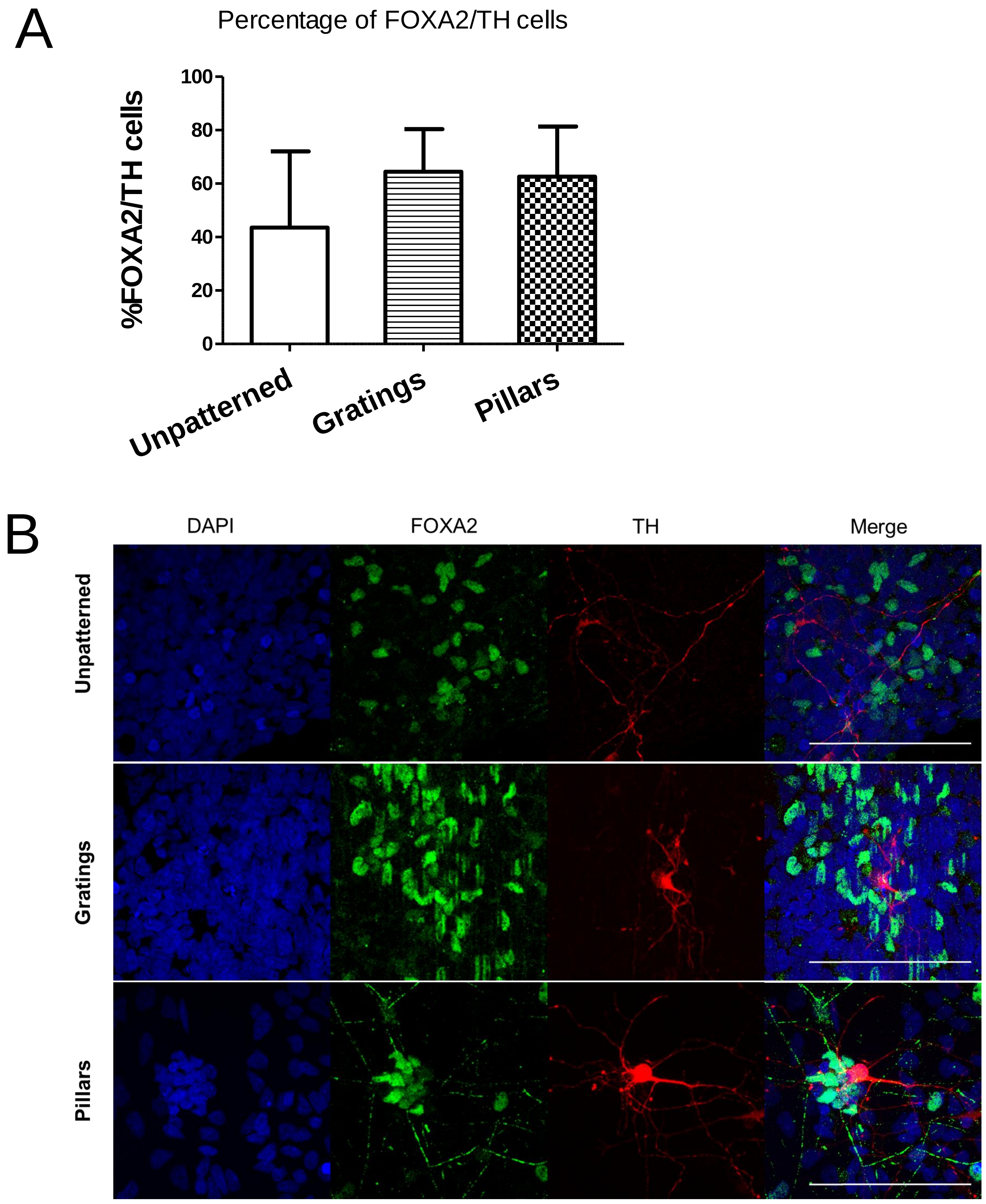

Supplementary Figure 5. (A) Quantification and data analysis of FOXA2 and TH co-expressing cells over TH cells after 21 day differentiation on unpatterned, gratings and pillars PDMS substrates. Data are represented as data ± SEM of 3 independent experiments. (B) Immunostaining of dopaminergic neuronal markers on human induced pluripotent stem cell (iPSC)-derived cells on unpatterned, gratings, and pillars PDMS substrates. Scale bar, 100 μm.

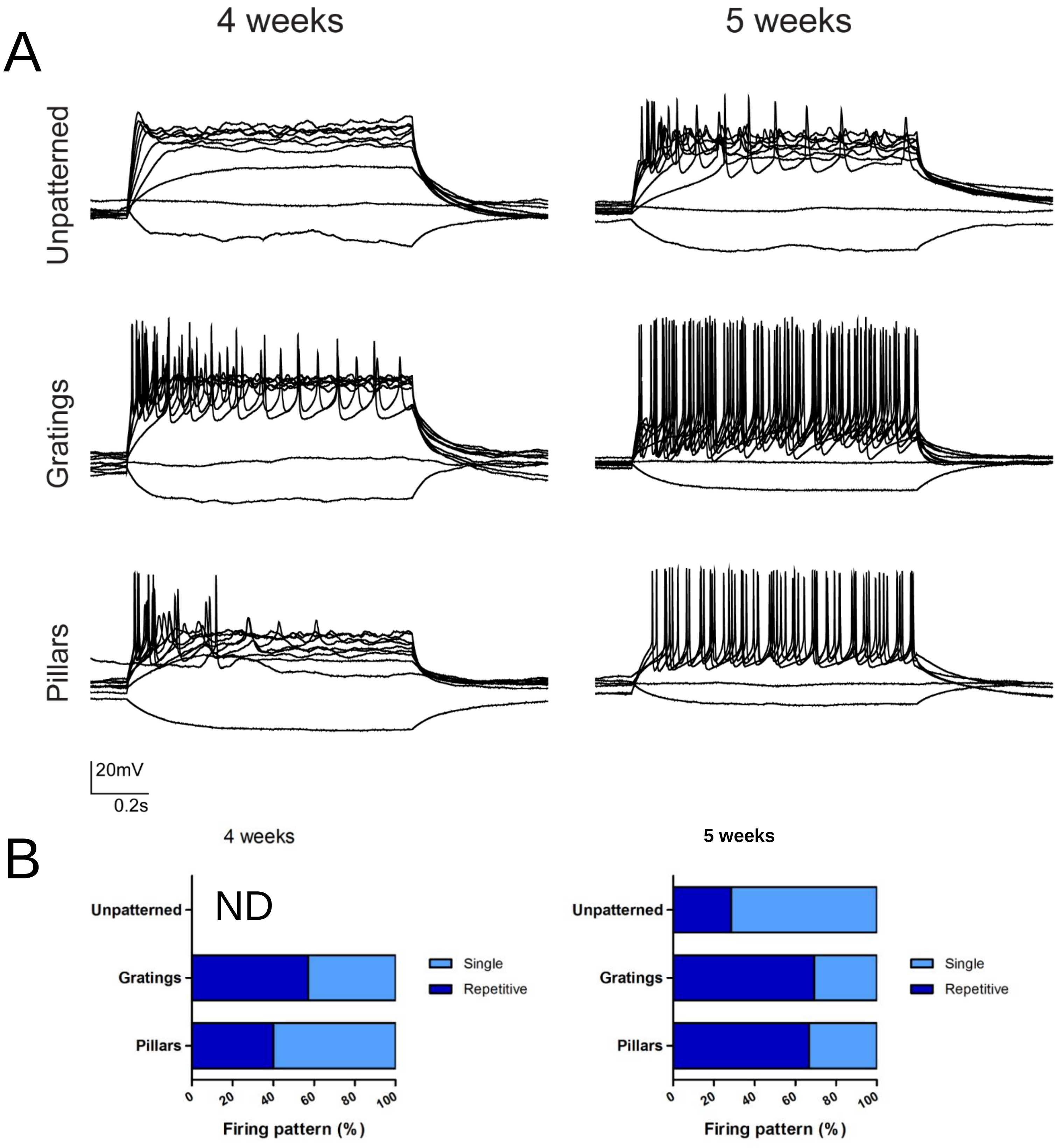

Supplementary Figure 6. Electrophysiology properties of human induced pluripotent stem cell (iPSC)-derived cells from PD patient with LRRK2 mutation on unpatterned, gratings, and pillars PDMS substrates. (A) Representative tracings of firing patterns on substrates at 4 and 5 weeks post-differentiation. Neurons were capable of firing repetitive action potential as a response to current injection as early as 4 weeks post differentiation. (B) Percentage of differentiated cells capable of repetitive firing at 5 weeks post differentiation. More neurons on gratings and pillars were capable of repetitive firing than unpatterned.
